# An Estimation of Private Household Costs to Receive Free Oral Cholera Vaccine in Odisha, India

**DOI:** 10.1371/journal.pntd.0004072

**Published:** 2015-09-09

**Authors:** Vittal Mogasale, Shantanu K. Kar, Jong-Hoon Kim, Vijayalaxmi V. Mogasale, Anna S. Kerketta, Bikash Patnaik, Shyam Bandhu Rath, Mahesh K. Puri, Young Ae You, Hemant K. Khuntia, Brian Maskery, Thomas F. Wierzba, Binod Sah

**Affiliations:** 1 International Vaccine Institute (IVI), Seoul, Republic of Korea; 2 Regional Medical Research Center (RMRC), Bhubaneswar, Odisha, India; 3 Directorate of Health Services (DHS), Odisha, India; Institute of Tropical Medicine, BELGIUM

## Abstract

**Background:**

Service provider costs for vaccine delivery have been well documented; however, vaccine recipients’ costs have drawn less attention. This research explores the private household out-of-pocket and opportunity costs incurred to receive free oral cholera vaccine during a mass vaccination campaign in rural Odisha, India.

**Methods:**

Following a government-driven oral cholera mass vaccination campaign targeting population over one year of age, a questionnaire-based cross-sectional survey was conducted to estimate private household costs among vaccine recipients. The questionnaire captured travel costs as well as time and wage loss for self and accompanying persons. The productivity loss was estimated using three methods: self-reported, government defined minimum daily wages and gross domestic product per capita in Odisha.

**Findings:**

On average, families were located 282.7 (SD = 254.5) meters from the nearest vaccination booths. Most family members either walked or bicycled to the vaccination sites and spent on average 26.5 minutes on travel and 15.7 minutes on waiting. Depending upon the methodology, the estimated productivity loss due to potential foregone income ranged from $0.15 to $0.29 per dose of cholera vaccine received. The private household cost of receiving oral cholera vaccine constituted 24.6% to 38.0% of overall vaccine delivery costs.

**Interpretation:**

The private household costs resulting from productivity loss for receiving a free oral cholera vaccine is a substantial proportion of overall vaccine delivery cost and may influence vaccine uptake. Policy makers and program managers need to recognize the importance of private costs and consider how to balance programmatic delivery costs with private household costs to receive vaccines.

## Author Summary

The price of vaccine and the costs of its delivery are two important economic measures considered by governments and various international organizations in their decisions on the use of a new vaccine. However, the costs to the vaccine recipients resulting from their travel, time and wage loss are hardly considered and rarely documented. Even if the vaccine is provided for free, the costs borne by vaccine recipients could be sufficient enough to be a hurdle for taking vaccine. We elucidate this less explored angle of “vaccine recipient cost” in the context of oral cholera vaccine mass campaign in Odisha, India. Our research shows that the potential loss of income for individuals for receiving oral cholera vaccine ranged from 25% to 38% of overall vaccine delivery costs. We believe our findings have global implications on future decisions and policy making on vaccine introduction in balancing programmatic delivery costs with private household costs to receive vaccines.

## Introduction

Several large cholera outbreaks in the sub Saharan Africa, Asia and Caribbean regions [1–3] have renewed interest in the use of oral cholera vaccines (OCV) in recent years. Considering the public health importance of cholera, the World Health Organization (WHO) recommends targeting OCVs to vulnerable populations living in high-risk areas in conjunction with other control measures [4]. The WHO prequalified OCV *Shanchol* is reported to confer 65% protective efficacy over five years against clinically-significant cholera [5].This vaccine has been used in several OCV mass campaigns worldwide in recent years [6–10] and available eligible countries through WHO stockpile [11].

When deploying OCVs during a vaccination campaign, budget-constrained public health staff will seek to minimize costs, particularly staff time, equipment, vaccine transport, and vaccine procurement costs. However, health staff may give less attention to the costs in transportation and lost wages incurred by individuals who seek vaccination. The travel and time costs borne by households are known to be crucial determinants in population-level access and uptake of vaccines. It has previously been reported that high out-of-pocket expenditure resulted in lower uptake in routine vaccination settings [12–16]. Similarly, higher indirect costs measured as travel distance and time has an adverse impact on vaccination coverage [16–19]. In Beira, Mozambique, the likelihood of participation and household cholera vaccine uptake was inversely related to travel costs [20]. Other than this Beira study, the household costs for OCV delivered through mass campaign settings has not been included in published estimates of vaccine delivery costs [8,21–24].

In this paper, we explore private household costs to receive free vaccine during an OCV campaign conducted in Odisha, India in 2011. These findings are potentially applicable to many other settings and vaccination programs.

## Methods

Based on a cross sectional survey, we estimated the private household costs for receiving OCV during a mass vaccination campaign conducted in Orissa, India in 2011. These costs included direct costs or out-of pocket expenses and indirect costs such as income loss due to the time spent for vaccination by the recipients and their caretakers.

### Direct Costs

There are two types of direct out-of pocket costs: medical and non-medical costs. The direct medical cost may include co-payments for vaccines or treatment for vaccine-related adverse events. There were no direct medical costs for this study because the vaccine was given free of cost and there were no adverse events following vaccination that required medical care [8]. Direct non-medical costs included transport costs from home to the vaccination site and were estimated based on a cross sectional survey described below.

### Indirect Costs

Indirect costs or costs related to income loss were estimated using a human capital approach based on the lost productivity of those vaccinated during the campaign [25]. In the human capital approach, the vaccine recipient perspective was taken and any hour not worked while receiving a vaccine was counted as hourly income loss. The income loss for adults was calculated by multiplying the self-reported participation time by daily wage rates. Wage loss was accounted for using two distinct methods: based on self-report by vaccine recipients (Method 1) and based on Odisha government minimum wage of INR 145 (USD 3.3) paid to semi-skilled individuals per day (Method 2) [26]. A third approach to measure productivity loss based on gross domestic products (GDP) per capita income per day (Method 3) was used in sensitivity analysis.

A minimum wage is the supportive lowest daily remuneration that employers may legally pay to workers for the skill category. Minimum wage is a conservative estimate. We weighed the minimum wage by age and job category. Many children do not earn wages but are often significant contributors to the economy and therefore their time should be monetised [27–29]. We applied age-specific wages separately for adults (15+ years); school aged children (5–14 years) and young children (1–4 years). Average hourly minimum wage was applied 100% for adults, 50% for school aged children and 25% for young children respectively [28,29]. To estimate the productivity loss from foregone non-market activities such as routine household chores, childcare, leisure time and school time, which is valued by the individuals, household and farm work was given 70% of daily/hourly wage, while leisure time was given 50% of daily/hourly wage [28,29].

For Method 3, we applied the GDP per capita in Odisha to value time. The time cost of each individual was valued equally irrespective of age or occupation. The state GDP per capita of Indian Rupee (INR) 53,578 (USD 1,205.5) was obtained from Indian Ministry of Statistics and Programme Implementation data for 2011–2012 [30]. Assuming 365 work days and 8 hours of productivity a day [31], estimated GDP-hour is translated to INR 146.79 (USD 3.30) per day or INR18.35 (USD 0.41) per hour. The assumption of 8 hours of productivity in GDP per capita estimation was used because non-market household production is usually not accounted in GDP estimation [32,33]. We did a sensitivity analysis where we assumed 24 hours of productivity a day in GDP estimation.

For conversion to USD from INR we used an exchange rate for April 1^st^ 2011 (1USD = 44.45 INR) based on Reserve Bank of India data [34] which is beginning of fiscal year [35].

### The Vaccination

The 2-dose OCV campaign was described in detail elsewhere [8]. In short, a baseline census was conducted from household surveys used to map the target population in the proposed vaccination areas of 10 health sub centers of Alagum community health center, Satyabadi block, Puri district, India. Subsequently, the OCV campaign was conducted at 62 vaccination booths located in the community, mostly at schools from May 5 to June 4, 2011. Schools were not in session at the time of vaccination campaign. Of the total 51,865 residents listed in the census, 31,552 eligible persons received the first dose of vaccine and 23,751 of these completed their second dose. This corresponds to coverage rates of 61% for the first dose and 46% for the second dose. The vaccine cost at market price was $1.85 and the public health vaccine delivery cost was $0.49 based on the review of project expenditure records and interview of key personnel [8]. GIS mapping of households and vaccination kiosks were used to quantify the physical distance between households and their nearest vaccination booths.

### The Survey

The direct out-of-pocket costs for travel to the vaccination site and the time in minutes waiting to receive vaccination was self-reported by recipients during a cross sectional survey. For the survey, nine villages were selected via stratified, simple random sampling. Villages were stratified by economic status (low-middle-high), location (more/less remote), size (number of households), and level of vaccine uptake (low-middle-high) during the campaign to give sufficient representation to these characteristics. All houses in the 9 selected villages were further stratified by 1) all or some household members taking two doses, 2) at least one household member receiving one dose, and 3) no household members receiving the vaccine. In total, 600 households, 200 from each of three categories (two doses, one dose and no dose) were randomly selected for conducting the socio-behavioral survey presented elsewhere [36]. The private costing questionnaire described here was administered to the subset of households that received one or two doses of vaccines only. For each household, one eligible individual (i.e., a permanent household member aged 18 years or older) was asked a series of questions regarding out-of-pocket and time costs for the last dose of vaccine they had received. The questions included the number of household members that received vaccines, travel cost, travel time, waiting time, loss of wages, what other activities they would have engaged in, the number of accompanying members, and income loss for accompanying members.

Double data entry was done in Microsoft FoxPro 7.0 (Microsoft, Seattle, WA, USA) and the data was analyzed using open source statistical software R [37]. All the costs presented here are incurred in year 2011 and presented in INR 2011 and USD 2011 without discounting. The GIS map and the distance between households and vaccination booth was estimated based on ArcGIS Desktop 9.3.1 (ESRI Redlands, CA, USA).

### Research Ethics

Written informed consent was obtained from the respondents. For participants unable to sign, a witness observed the consenting process and signed the consent form. The study protocol including consent process where witness observed the consenting process for participants unable to sign was approved by relevant ethical committees. This included Institutional Review Board of the International Vaccine Institute (IVI), Seoul, Korea (Ref. No: IVI IRB# 2010–003) and Human Ethical Committee of the Regional Medical Research Center (RMRC), Bhubaneswar, Odisha (letter dated 19th January 2010).

## Results

Among the 600 randomly selected households, five households could not be reached. Of the remaining households, the 337 households that reported one or more members received vaccine during the mass vaccination campaign were interviewed.

The most common occupation of the interviewed heads of households was farming (n = 122; 36.2%), followed by work with daily wage compensation (n = 63; 18.7%), trading (n = 40; 11.9%), unemployed (n = 27; 8%) and retired (n = 17; 5%). The majority of household heads were literate without formal education (n = 69; 20.5%) or attended primary school (n = 98; 29.1%). Some of them had secondary school (n = 62; 18.4%) or high school and higher education (n = 63; 18.7%) while, 13.4% (n = 45) household heads were illiterate. A majority of the interviewed households (n = 240; 71.2%) reported practicing open field defecation.

Seventy three percent (245/337) of the respondents were females, many men were at work. The mean age of female and male respondents were 37.0 (SD = 13.0) and 44.2 years (SD = 15.7) respectively. The mean number of members in interviewed households was 5.7 (SD = 2.8) of which on average 4.0 (SD = 2.3) household members were vaccinated with at least one dose. Nearly 70% of the vaccine recipients in the interviewed household were adults. The household demographic characteristics are presented in Table 1.

**Table 1:** Demographics characteristics of the surveyed households (N = 337).

Age group	< 5 yrs	5–14 yrs	>14 yrs	Total
Number of people in the households	110	356	1440	1,906
People who received at least one dose of vaccine	61	298	826	1,185
Percentage who received at least one dose of vaccine	55.5%	83.7%	57.4%	62.2%
Number of females in the households	45	159	739	943
Females who received at least one dose of vaccine	23	138	456	617
Percentage of females received at least one dose of vaccine	51.1%	86.8%	61.7%	65.4%

**Fig 1:**
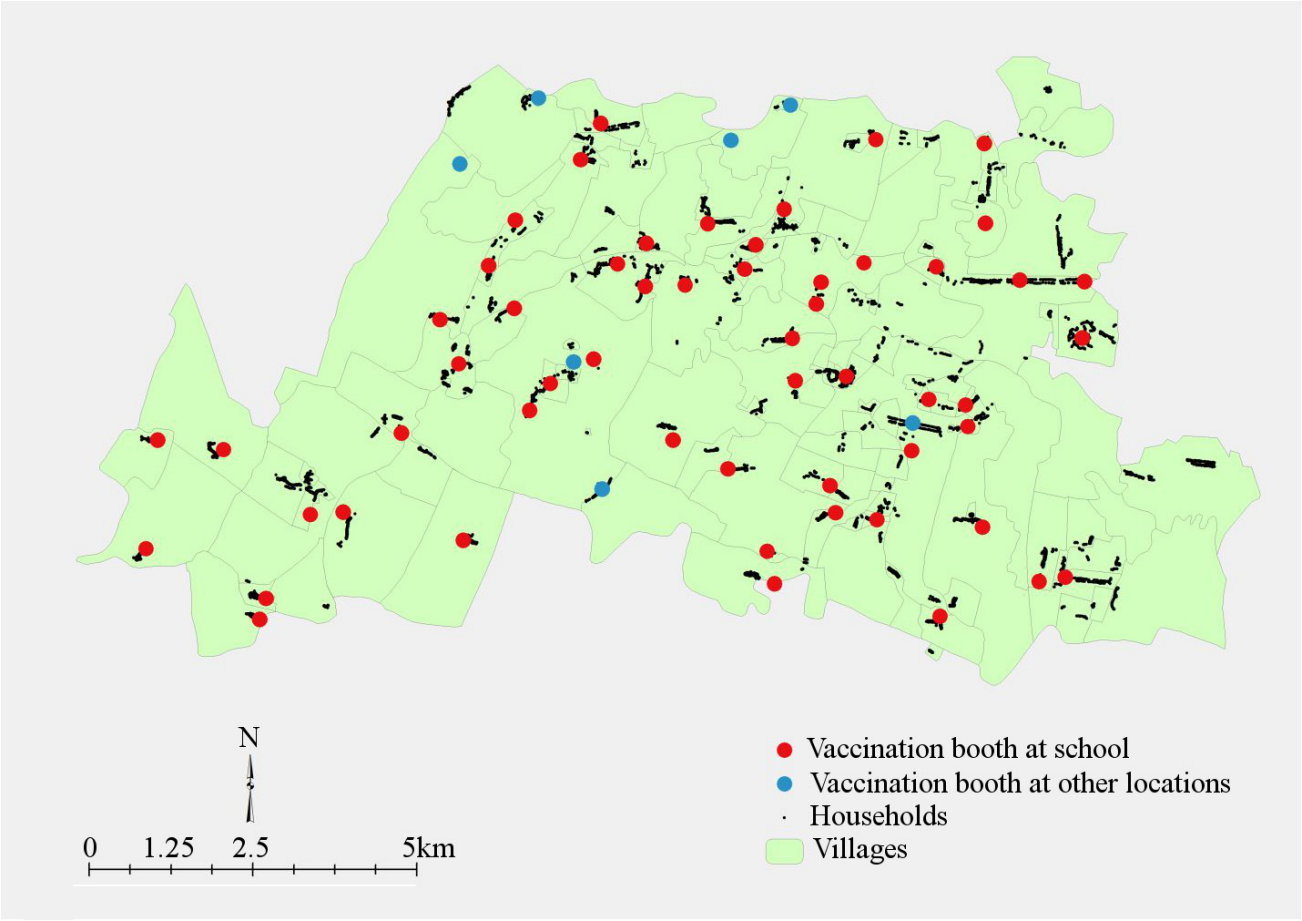
Geographical information system map indicating location of households and vaccination booths in 10 sub centers of Satyabadi block, Puri district, India.

Most households were located adjacent to the vaccination booths (Fig 1) with an average distance of 282.7 meters from nearest vaccination booth (SD = 254.5, median 202.6, minimum 3.2, maximum 1,303). The average distance to the nearest vaccination booth in interviewed households (N = 337) was lower than the average distance to nearest vaccination booth in all vaccinated households (N = 9,166; mean = 311.8, SD = 240.5, p value = 0.03) in the study site. This distance between households and vaccination booths was calculated from GIS maps, and does not account for physical barriers such as absence of road, ponds, rivers, and lakes between houses and booths.

Most household members walked from their homes to vaccination booths (92%; n = 309/337). The remainder used bicycles (10%; n = 33/337) or motorbikes (2%; n = 7/337), and one household used a car. The total percentage is over 100 as some households reported using more than one mode of transport. Interviewed people reported an average of 26.5 minutes of travel time to reach vaccination booths and a waiting time of 15.7 minutes to receive vaccination (Table 2). Some individuals spent up to 3 hours travelling and up to 2 hours waiting for vaccination. Around one half of the people in households missed a part of their work time to receive the vaccination (Table 2).

**Table 2:** Productivity loss for receiving vaccines.

Item	Productivity loss per household			Productivity loss per person		
	Mean (SD)	Median	Range	Mean (SD)	Median	Range
Travel time (minutes)	106.58 (118.87)	80	0–1170	26.46 (22.36)	20	0–180
Waiting time (minutes)	65.05 (85.63)	40	0–780	15.67 (16.73)	10	0–120
Number of people who missed paid work/1000 doses	166.17 (513.78)	0	0–4000	43.05 (138.12)	0	0–1000
Number of people who missed household work or farm work/1000 doses	302.67 (785.14)	0	0–6000	80.32 (205.88)	0	0–1000
Number of people who missed school/college/1000 doses	11.87 (153.84)	0	0–2000	2.37 (30.77)	0	0–400

The out-of-pocket travel cost per family for receiving vaccination was negligible (INR 0.68; USD 0.02). The productivity loss depended on the calculation method (Table 3). The productivity loss estimated based on minimum wages (Method 2) was least, followed by estimation based on reported potential loss of income (Method 1) while, productivity loss based on GDP (Method 3) was almost twice that of the former two methods. If 24 hours of productivity was assumed in estimating GDP per capita, the productivity loss in Method 3 was least at $0.09 ($0.0 to $0.62).

**Table 3:** Private costs to receive one dose of cholera vaccine in Odisha India in USD.

Item	Cost per family for one round			Cost per person per dose			Mean cost per person per dose as % minimum daily wage
	Mean (SD)	Median	Range	Mean (SD)	Median	Range	
Private out of pocket cost (for travel)	0.02 (0.15)	0	0.0–2.27	0.009 (0.13)	0	0.0–2.27	0.27%
Indirect cost- as per reported wage loss (Method 1)	0.72 (3.23)	0	0.0–44.99	0.18 (0.83)	0	0.05	5.52%
Indirect cost-based on minimum wage (Method 2)	0.62 (0.64)	0.44	0.0–6.12	0.15 (0.12)	0.12	0.0–1.08	4.60%
Indirect cost- based on GDP per capita (Method 3)	1.18 (1.21)	0.88	0.0–13.42	0.29 (0.20)	0.24	0.0–1.86	8.89%

The private cost of receiving oral cholera vaccine was 24.6%–38.0% of the total vaccine delivery costs depending on the method used to value vaccine recipients’ productivity loss (Table 4). If 24 hours of productivity was assumed in GDP calculation, the private cost of receiving oral cholera vaccine was 17.0% of vaccine delivery costs.

**Table 4:** Private costs as a percentage of overall vaccine delivery/receipt cost.

Cost items	Cost in USD		
	Method 1*	Method 2*	Method 3*
Public health vaccine delivery cost [8]	0.49 (72.1%)	0.49 (75.4%)	0.49 (62.0%)
Private out of pocket cost (for travel)	0.01 (1.5%)	0.01 (1.5%)	0.01 (1.3%)
Private productivity loss	0.18 (26.5%)	0.15 (23.1%)	0.29 (36.7%)
Total vaccination cost	0.68 (100%)	0.65 (100%)	0.79 (100%)

*Method 1: indirect cost as per reported wage loss

Method 2: indirect cost based on minimum wage

Method 3: indirect cost based on GDP per capita

## Discussion

Our analysis shows that private cost, i.e., direct travel costs and indirect productivity losses during the Odisha mass campaign ranged from 0.16 to 0.30 USD per dose (0.62 to 1.18 USD per family). This is the marginal costs to vaccine recipients, despite the vaccination booths were organized close proximity to the households. Although the vaccine was provided for free during the campaign, vaccine recipients and those who accompanied them had to forego time and money. This indicates a need for operational approaches and robust planning to reduce private household costs in future OCV campaigns and potentially in other vaccination programs.

In recent years, there have been several recommendations for approaches to reduce out of pocket expenditure and improve vaccination uptake in routine settings [15,38,39]. As such, an OCV campaign has distinct challenges related to private costs- unlike most childhood vaccines. The OCV is targeted to populations greater than one year of age (except for pregnant women), of whom a large proportion may be working men and women. High coverage rates among working men and women are difficult to achieve because of their greater opportunity costs relative to children. Although approaches such as house-to-house vaccine delivery have helped to achieve nearly universal coverage of the oral polio vaccine among children [40], such approaches may not produce similar coverage rates in OCV campaigns since adults may be unreachable during work days. Moreover, house-to-house visits are more costly for service providers and logistically challenging. In Odisha, there were no out-of-pocket payments required to receive OCVs, and extra efforts were made to set-up vaccination booths close to households [8] so that private travel costs could be minimized. Despite these measures, the productivity losses were nearly one third of total vaccine delivery costs due to potential or forgone income loss. The vaccination booths operated from 7 am to 5 pm which fell during working hours for many participants. People then had to choose between receiving the vaccine and performing their routine activities based on their perceived benefits. Modified approaches such as flexible location and time may suit the work-leisure patterns of local populations and may be beneficial to reduce private vaccination costs in future programs. Similarly, it is valuable to have context and vaccine specific approaches that can reduce private household costs and improve vaccination coverage.

Our finding on the need to reduce private costs for receiving OCV is applicable to other routine vaccination programs. As described in the introduction high out of pocket expenditures and indirect costs measured by travel distance and time adversely affect routine vaccine uptake [12–19]. Program managers should consider developing site specific approaches to reduce private costs as vaccine recipients costs are under recognized in program planning and implementation.

There are certain limitations of this research owing to study design and study timing. The indirect cost is a conservative estimate as the average distance to vaccination booth from sampled households was lower than average distance to vaccination booth from all vaccinated households (282.7 meters vs. 311.8 meters). Assuming the same travel time per meter among sampled population and all vaccinated population, the travel distance of 311.8 meters would have taken 29.18 minutes per dose instead of 26.46 estimated in the sampled population. Thus the time cost is underestimated. Reported out-of-pocket costs and waiting time were collected nearly four months after vaccination and subject to recollection and reporting biases commonly encountered in population based surveys. Moreover, the participant time cannot be directly valued in the marketplace, especially for women and children not attending school. This leads to imprecision regardless of the method used to value their time.

### Conclusion

Oral cholera mass vaccination involves costs related to productivity loss to vaccine recipients which may adversely influence vaccination decision. Locally appropriate programmatic approaches are necessary to reduce time and costs involved in receiving OCV. Future program planning and vaccination costing studies should account for costs to service provider as well as service recipients. Global, regional and country level decision makers as well as local program managers should account for the potential implications of private household costs on coverage levels while deploying new vaccines and consider the costs to recipients as important as the cost to providers.

## Supporting Information

S1 Checklist STROBE statement checklist. (DOCX)
