# Supplementary material for: An Estimation of Private Household Costs to Receive Free Oral Cholera Vaccine in Odisha, India
Source: PLoS Negl Trop Dis. 2015 Sep 9;9(9):e0004072. doi: 10.1371/journal.pntd.0004072 (PMC4564266; doi:10.1371/journal.pntd.0004072)
Supplement: S1 Checklist — (DOCX) [file pntd.0004072.s001.docx]

STROBE Statement—checklist of items that should be included in reports of observational studies

|  | | Item No. | | Recommendation | Page  No. | | Relevant text from manuscript |
| --- | --- | --- | --- | --- | --- | --- | --- |
| **Title and abstract** | | 1 | | (*a*) Indicate the study’s design with a commonly used term in the title or the abstract | 2 | | Cross-sectional survey |
|  |  |  |  | (*b*) Provide in the abstract an informative and balanced summary of what was done and what was found | 2 | | Given under methods, findings and interpretation (line 42-62) |
| Introduction | | | | | | |  |
| Background/rationale | | 2 | | Explain the scientific background and rationale for the investigation being reported | 4 | | Line 87-98 describes rationale |
| Objectives | | 3 | | State specific objectives, including any prespecified hypotheses |  | | 100-101 provides objectives |
| Methods | | | | | | |  |
| Study design | | 4 | | Present key elements of study design early in the paper | 5 | | 106-109 key elements |
| Setting | | 5 | | Describe the setting, locations, and relevant dates, including periods of recruitment, exposure, follow-up, and data collection | 6-7 | | 155-186 describes all points |
| Participants | | 6 | | (*a*) *Cohort study*—Give the eligibility criteria, and the sources and methods of selection of participants. Describe methods of follow-up  *Case-control study*—Give the eligibility criteria, and the sources and methods of case ascertainment and control selection. Give the rationale for the choice of cases and controls  *Cross-sectional study*—Give the eligibility criteria, and the sources and methods of selection of participants | 7 | | Cross sectional survey Line 171-183 |
|  |  |  |  | (*b*) *Cohort study*—For matched studies, give matching criteria and number of exposed and unexposed  *Case-control study*—For matched studies, give matching criteria and the number of controls per case |  | | Not applicable |
| Variables | | 7 | | Clearly define all outcomes, exposures, predictors, potential confounders, and effect modifiers. Give diagnostic criteria, if applicable | 5-6 | | Line 111-153, direct cost, indirect costs and estimation methods |
| Data sources/ measurement | | 8* | | For each variable of interest, give sources of data and details of methods of assessment (measurement). Describe comparability of assessment methods if there is more than one group |  | | Provided. Ref. no 25-35 |
| Bias | | 9 | | Describe any efforts to address potential sources of bias | 5,11 | | Line 124-128 talks how reporting bias was addressed using sensitivity analysis. Line 311-313 explains the bias |
| Study size | | 10 | | Explain how the study size was arrived at | 7 | | Line 175-179 |
| Continued on next page Quantitative variables | 11 | | Explain how quantitative variables were handled in the analyses. If applicable, describe which groupings were chosen and why | | 5-6 | Line 120-150 explains how variables were handled | |
| Statistical methods | 12 | | (*a*) Describe all statistical methods, including those used to control for confounding | | 7 | Line 188-192 explains analysis | |
|  |  |  | (*b*) Describe any methods used to examine subgroups and interactions | |  | No subgroup analysis was done | |
|  |  |  | (*c*) Explain how missing data were addressed | |  | Had no missing data | |
|  |  |  | (*d*) *Cohort study*—If applicable, explain how loss to follow-up was addressed  *Case-control study*—If applicable, explain how matching of cases and controls was addressed  *Cross-sectional study*—If applicable, describe analytical methods taking account of sampling strategy | |  | Not applicable | |
|  |  |  | (*e*) Describe any sensitivity analyses | | 5-6 | Line 120-150 describes sensitivity analysis | |
| Results | | | | | | | |
| Participants | 13* | | (a) Report numbers of individuals at each stage of study—eg numbers potentially eligible, examined for eligibility, confirmed eligible, included in the study, completing follow-up, and analysed | | 8 | Line 206-208 | |
|  |  |  | (b) Give reasons for non-participation at each stage | | 8 | Line 206-208 | |
|  |  |  | (c) Consider use of a flow diagram | |  | Very simple and did not need flow diagram | |
| Descriptive data | 14* | | (a) Give characteristics of study participants (eg demographic, clinical, social) and information on exposures and potential confounders | | 8,16 | Line 210-223,table 1 | |
|  |  |  | (b) Indicate number of participants with missing data for each variable of interest | |  | Not applicable | |
|  |  |  | (c) *Cohort study*—Summarise follow-up time (eg, average and total amount) | |  |  | |
| Outcome data | 15* | | *Cohort study*—Report numbers of outcome events or summary measures over time | |  |  | |
|  |  |  | *Case-control study—*Report numbers in each exposure category, or summary measures of exposure | |  |  | |
|  |  |  | *Cross-sectional study—*Report numbers of outcome events or summary measures | | 9,16,17 | Line 238-256; Table 2 and 3 | |
| Main results | 16 | | (*a*) Give unadjusted estimates and, if applicable, confounder-adjusted estimates and their precision (eg, 95% confidence interval). Make clear which confounders were adjusted for and why they were included | |  | Not applicable | |
|  |  |  | (*b*) Report category boundaries when continuous variables were categorized | | 9,16,17 | Line 238-256; Table 2 and 3 | |
|  |  |  | (*c*) If relevant, consider translating estimates of relative risk into absolute risk for a meaningful time period | |  | Not relevant | |

Continued on next page

| Other analyses | 17 | Report other analyses done—eg analyses of subgroups and interactions, and sensitivity analyses | 9,16,17 | Line 238-263; Table 2,3 and 4 |
| --- | --- | --- | --- | --- |
| Discussion | | | | |
| Key results | 18 | Summarise key results with reference to study objectives | 10 | Line 270-276 |
| Limitations | 19 | Discuss limitations of the study, taking into account sources of potential bias or imprecision. Discuss both direction and magnitude of any potential bias | 11 | Line 305-315 |
| Interpretation | 20 | Give a cautious overall interpretation of results considering objectives, limitations, multiplicity of analyses, results from similar studies, and other relevant evidence | 11 | Line 318-320 |
| Generalisability | 21 | Discuss the generalisability (external validity) of the study results | 11,12 | Line 320-325 |
| Other information | |  | | |
| Funding | 22 | Give the source of funding and the role of the funders for the present study and, if applicable, for the original study on which the present article is based |  | Disclosed separately |

*Give information separately for cases and controls in case-control studies and, if applicable, for exposed and unexposed groups in cohort and cross-sectional studies.

**Note:** An Explanation and Elaboration article discusses each checklist item and gives methodological background and published examples of transparent reporting. The STROBE checklist is best used in conjunction with this article (freely available on the Web sites of PLoS Medicine at http://www.plosmedicine.org/, Annals of Internal Medicine at http://www.annals.org/, and Epidemiology at http://www.epidem.com/). Information on the STROBE Initiative is available at www.strobe-statement.org.
